# Neuropathic Pain Related to Peripheral Neuropathies According to the IASP Grading System Criteria

**DOI:** 10.3390/brainsci11010001

**Published:** 2020-12-22

**Authors:** Giulia Di Stefano, Andrea Di Lionardo, Giuseppe Di Pietro, Andrea Truini

**Affiliations:** Department of Human Neuroscience, Sapienza University of Rome, Viale Università, 30-00185 Rome, Italy; andrea.dilionardo@uniroma1.it (A.D.L.); giuseppe.dipietro@uniroma1.it (G.D.P.); andrea.truini@uniroma1.it (A.T.)

**Keywords:** neuropathic pain, grading system, ongoing pain, paroxysmal pain, allodynia

## Abstract

Neuropathic pain is defined as pain caused by a lesion or disease of the somatosensory system. Neuropathic pain represents a broad category of pain conditions, common complications of peripheral neuropathies, which are characterized by a combination of positive symptoms, including paresthesia and/or dysesthesia and sensory deficits in the painful area. In the present paper, we aimed to assess neuropathic pain frequency and clinical characteristics of peripheral neuropathies due to different aetiologies according to grading system criteria of the International Association for the Study of Pain for a definitive diagnosis of neuropathic pain. Epidemiological studies applying these criteria have been conducted in patients with diabetes, brachial plexus injury, and other traumatic nerve injuries. Neuropathic pain was diagnosed in 37–42% of patients with diabetic peripheral neuropathy, 56% of patients with brachial plexus injury, and 22% of patients with intercostobrachial neuropathy. The most frequent neuropathic pain type was ongoing pain (described as burning or pressing), followed by paroxysmal pain (electric shock-like sensations) and allodynia (pain evoked by brushing and pressure). By providing information on the frequency, clinical signs, and variables associated with neuropathic pain due to different aetiologies, these studies contribute to improving the clinical management of this condition.

## 1. Introduction

The term neuropathic pain (NP) refers to the pain arising as a direct consequence of a lesion or disease affecting the somatosensory system [1]. According to epidemiological studies, the prevalence of NP in the general population is 7–8%, which accounts for 20–25% of individuals with chronic pain [2].

NP represents a major issue in many peripheral nervous system neuropathies, especially in distal symmetrical peripheral neuropathies (such as diabetic polyneuropathy) and focal neuropathies following traumas (such as traumatic brachial plexus lesions) or surgical procedures (such as breast surgery). The International Association for the Study of Pain (IASP) grading system represents a tool to ascertain the level of certainty with which pain in a particular peripheral nerve disorder can be classified as neuropathic [1,3] considering medical history, clinical examination showing negative or positive sensory signs with a plausible neuroanatomical distribution, and confirmatory diagnostic tests.

NP syndromes are clinically characterized by a combination of positive and negative signs and may manifest with a constellation of different symptoms, including ongoing burning pain, pressing pain, paroxysmal electric shock-like sensations or stabbing pain, and dynamic mechanical allodynia [4]. Different mechanisms determine each kind of symptoms. Knowledge of these pathophysiological mechanisms should guide the specific treatment strategy [4]. Using a mechanism-based approach, the IASP grading system algorithm could help identify a subset of patients who may respond to specific treatment strategies.

According to the revised version of the IASP grading system [3], sensory deficits are not a prerequisite for all NP conditions. In patients with peripheral nerve lesions or hereditary channelopathies, touch-evoked allodynia or thermal hyperalgesia may be present even without detectable sensory deficits. When sensory signs are hard to detect, but the nature of the lesion is known and diagnostic tests confirm the impairment of the somatosensory nervous system, the level “probable” is still applicable. Various tests, including nerve conduction study, quantitative sensory testing, evoked potentials, neuroimaging, and skin biopsies, can be used to identify a lesion affecting the somatosensory system. However, these tests are available only in specialized centers; moreover, they do not measure pain per se and cannot directly confirm the causal relationship between the detected somatosensory system lesion and pain. For this reason, NP diagnosis is still dependent on clinical judgment and the interpretation of diagnostic tests in a specialized clinical context.

In randomized controlled trials, pain type was often unspecified, and patients with both nociceptive and NP were considered equally in the analyses. Few studies have clearly stated whether only patients with pain distributed in a body territory with sensory signs corresponding to a somatosensory system lesion were included. Moreover, only a limited number of trials applied diagnostic procedures, including quantitative sensory testing or pain-related evoked potentials. This heterogeneity in patient samples probably led to a significant number of negative or uncertain and conflicting results. The IASP grading system could be used to reduce heterogeneity in clinical trials testing NP treatment efficacy and to improve the accuracy of effect estimates.

We carried out a systematic literature search, aiming to determine the frequency and clinical characteristics of NP diagnosed according to the new IASP grading system in patients with peripheral neuropathy due to different aetiologies.

## 2. Search Strategy

In the literature search process, we referred to PubMed, EMBASE, and the Cochrane Database of Systematic Reviews and considered publications between January 2008 and September 2020. Search terms included NP frequency and IASP grading system criteria. The first search phase was followed by a second one, referring to the bibliographies of the retrieved articles. Only full-length original manuscripts were considered, and the search was limited to English language publications. Two reviewers independently carried out the review process, and only publications approved by both of them were taken into account. The reviewers independently assessed the quality of the studies. We included studies involving patients with peripheral neuropathies due to different aetiologies and a diagnosis of definite or probable NP according to IASP grading system criteria. The analysis focused on NP clinical characteristics and frequency in patients with peripheral neuropathy of different aetiologies.

## 3. Results

Forty-seven studies were identified. After abstract screening, 23 full texts were assessed for eligibility. We excluded 13 studies that were not relevant or that did not apply the IASP grading system criteria. We included nine studies assessing patients with diabetic peripheral neuropathy [5,6], traumatic brachial plexus injury [7], postsurgical pain [8,9,10], or other peripheral NP conditions [11,12,13] (Table 1). In the nine included studies, NP diagnosis was consistent with both the old and the revised grading system criteria.

### 3.1. Diabetic Peripheral Neuropathy

Large epidemiological studies tested the prevalence of NP in diabetic patients. In a cross-sectional study involving 766 diabetic patients, the prevalence of chronic pain with neuropathic characteristics was 20.3% [14]. An observational study was conducted on a wide cohort of diabetic patients receiving community-based healthcare in northwest England (*n* = 15,692). The prevalence of painful symptoms was 34%, while the prevalence of painful diabetic neuropathy was 21% [15]. However, these studies did not use diagnostic tests that could provide definitive evidence of peripheral nerve damage and did not provide information on small fiber impairment.

The Neuropathic Pain Special Interest Group (NeuPSIG) of the Italian Neurological Society (SIN) conducted a large epidemiological study to investigate the frequency and clinical characteristics of NP in patients with diabetic polyneuropathy [5]. A total of 816 patients who visited seven Italian diabetic outpatient clinics were consecutively enrolled. The definitive diagnosis of mixed or small fiber polyneuropathy was based on clinical signs, standard neurophysiological tests, and morphometric study [16] or quantitative sensory test findings [17,18]. The authors investigated autonomic symptoms through a systematic interview focusing on cardiovascular, genitourinary, and gastrointestinal disturbances.

Patients were definitively diagnosed with NP when examination excluded other causes of pain, pain had a neuroanatomical distribution confirmed by clinical signs, a score ≥4 was obtained on the DN4 questionnaire, and diagnostic test abnormalities indicated mixed or small fiber polyneuropathy. A definitive diagnosis of mixed or small fiber polyneuropathy was obtained in 36% and 2.5% of diabetic patients, respectively. The frequency of polyneuropathy was higher in patients with type 2 as opposed to type 1 diabetes (38% vs. 23%). Patients with mixed fiber polyneuropathy were older, had a longer diabetes duration, and had higher HbA1c and a higher body mass index than patients without polyneuropathy. Mixed fiber involvement was associated with microangiopathic diabetic complications, such as nephropathy, retinopathy, and arterial hypertension. In contrast, small fiber neuropathy was not associated with the main clinical variables, thus suggesting that other specific factors, including voltage-gated sodium channel variations, may increase the risk of small fiber impairment [19,20]. This finding is consistent with evidence of *SCN9A* variants in painful diabetic neuropathy [21,22]. Out of 816 diabetic patients (123 with type 1 and 693 with type 2 diabetes) enrolled in the Italian epidemiological study, 115 (13%) had painful polyneuropathy. The most representative pain quality was ongoing burning pain. Females with mixed fiber and patients with small fiber polyneuropathy had a higher risk of NP. As previously hypothesized, these findings might be due to greater pain sensitization and reporting due to specific hormonal and/or psychosocial factors [23].

Autonomic symptoms were reported by 9% of the patients. In a recent study involving 133 consecutive patients with diabetic polyneuropathy, NP frequency in pure large fiber polyneuropathy was similar to that observed in mixed or small fiber polyneuropathy, thus suggesting that nociceptive nerve terminal involvement might not be detected by standard diagnostic techniques (laser-evoked potentials and skin biopsy) [24]. This finding suggests the possible use of autonomic function tests (AFTs) as additional diagnostic tools for the assessment of small fiber function. Future studies should focus on the correlation between autonomic dysfunction and small fiber impairment in painful diabetic neuropathy and on the usefulness of AFTs as diagnostic tools. An accurate evaluation of the autonomic function may be obtained by a combination of cardiovascular autonomic and sudomotor function tests assessed by the composite autonomic scoring scale. Moreover, the assessment of sweat gland nerve fiber density may be a structural marker of sudomotor dysfunction.

A recent cross-sectional study involving 389 Danish patients with type 2 diabetes investigated the occurrence of peripheral neuropathy and NP [6]. Patients underwent clinical examination focused on sensory profiles, quantitative sensory testing, nerve conduction study, and morphometric study. According to standardized criteria, 126 patients (32%) had definite diabetic peripheral neuropathy. Of these, 53 (14%) had painful diabetic peripheral neuropathy.

### 3.2. Traumatic Brachial Plexus Injury

Several epidemiological studies investigating pain in patients with brachial plexus lesions did not use validated screening tools or investigate the different qualities of NP [25,26,27,28].

The NeuPSIG conducted a multicenter epidemiological study to investigate NP frequency and clinical features in patients with brachial plexus injury [7]. A total of 107 patients with traumatic brachial plexus lesions were enrolled at five Italian hospitals. A definitive diagnosis of traumatic brachial plexus injury was based on clinical and neurophysiological criteria according to recommendations established by the American Academy of Neurology guidelines [29]. Patients were grouped according to the clinically documented presence or absence of NP according to the DN4 questionnaire [30]. Patients with NP completed the Neuropathic Pain Symptom Inventory (NPSI) questionnaire. Subscores for spontaneous, paroxysmal, and provoked pain and abnormal sensations were calculated [31]. Sixty out of 107 patients (56%) experienced NP. Patients with NP had lower sensory nerve action potential amplitude than those without, suggesting that NP is related to peripheral nerve damage severity. The mean severity of the different pain qualities, calculated according to an 11-point numerical rating scale, was 4.9 ± 2.6 for burning pain, 4.7 ± 3.4 for pain resembling pins and needles, and 4.6 ± 3.4 for electric shock-like pain. The most frequent and severe pain quality was ongoing burning pain. This finding reflects the peculiar peripheral nerve damage related to brachial plexus injury. These lesions affect the cell body or postganglionic axon [4,32] and leave the second-order postsynaptic membrane exposed to local transmitters, giving rise to abnormal spontaneous firing.

### 3.3. Postsurgical Pain

Haroutiunian and colleagues performed a systematic literature review and selected 281 studies involving patients with persistent postsurgical pain after 11 different surgical procedures [8]. The prevalence of NP in each surgical group was determined by applying the IASP grading system criteria. A definitive diagnosis of NP was based on history of previous surgery, pain with neuroanatomically plausible distribution, demonstration of neuroanatomical distribution (presence of sensory disturbances concordant with pain distribution), and nerve lesions by confirmatory tests. The prevalence of definite NP in patients with persistent postsurgical pain was 68% after thoracic or breast surgical procedures, 31% after groin hernia repair, and 6% after hip or knee arthroplasty. This variability among surgical procedures probably depends on the likelihood of surgical iatrogenic nerve injury.

A longitudinal study involving 40 patients who previously underwent breast cancer surgery assessed the prevalence, clinical characteristics, and course of intercostobrachial neuropathy with or without NP [9]. Intercostobrachial neuropathy was detected in 23 patients (57.5%). Five patients (13%) experienced NP according to neurological examination and the DN4 questionnaire. While sensory disturbances improved during the one-year observation, NP did not. NPSI analysis showed that dynamic mechanical allodynia and burning pain were the most frequent pain types.

Høimyr and colleagues investigated the prevalence and severity of pain after lymph node excision in 178 patients with melanoma [10]. Eighty-one patients (47%) reported sensory changes, and 34 patients (19.7%) experienced postsurgical pain. Pain was more frequent after lymph node dissection (34%) than after sentinel node biopsy (14%). Pain was described as burning, pressing, and evoked by light pressure or touch (dynamic mechanical allodynia). In this study, sensory abnormalities were the most important predictor of pain. Twelve patients were enrolled in a follow-up study, and 10 of them fulfilled the grading system criteria for probable NP.

### 3.4. Pain in Different Neurological Disorders

NP prevalence was investigated in 120 patients with several chronic non-malignant pain conditions, including cervicobrachial, hip, knee, shoulder, arm, hand, and ankle pain and plexus brachialis lesions [11]. Twenty-two patients (18.3%) were classified as having probable or definite NP, while 90 patients (75%) were classified as having unlikely NP. NP prevalence according to the IASP grading system criteria was also assessed in patients with neck/upper limb pain, and definite NP was diagnosed in 45 out of 152 patients (30%) [12].

An epidemiological cross-sectional study applying the IASP grading system criteria provided data from 2173 patients [13]. Patients suffering from any pain condition who received treatment at a pain clinic were enrolled. Definite and probable NP were identified in 639 (29.4%) and 304 (14.0%) patients, respectively. Pure NP was diagnosed in 344 (15.8%) patients.

## 4. Discussion

NP is a major burden in patients with peripheral neuropathy due to various aetiologies. According to recent epidemiological studies, NP was diagnosed in 37–42% of patients with diabetic peripheral neuropathy, 56% of patients with brachial plexus injury, and 22% of patients with intercostobrachial neuropathy.

We performed a systematic literature review on the frequency and clinical features of NP related to peripheral neuropathies according to the revised IASP grading system criteria. The lack of valid diagnostic tests to diagnose and quantify NP requires a grading system based on judgment. Whereas the grade “possible” can be considered as a working hypothesis, the grades “probable” and “definite” require confirmatory evidence from a neurologic examination. The inclusion of patients with peripheral neuropathies due to different aetiologies and a diagnosis of definite or probable NP improved the certainty on the neuropathic origin of the pain symptoms.

The inclusion of studies based on the IASP grading system algorithm limited the present analysis to a small subset of peripheral nervous system diseases, thus not allowing the analysis of the full NP spectrum. However, this approach reduced the methodological heterogeneity among studies and provided information about the clinical characteristics and sensory profiles underlying different pathophysiological mechanisms [4].

Regardless of the aetiology, the most representative NP qualities include ongoing pain (burning and pressure pain), paroxysmal pain (electric shock-like sensations) and allodynia (pain elicited by brushing and pressure). These pain qualities result from different pathophysiological mechanisms.

In patients with distal symmetrical peripheral neuropathy, such as diabetic polyneuropathy, ongoing burning pain arises from the hyperexcitability of irritable nociceptors or regenerating nerve sprouts [4,33]. In the study by Truini and colleagues on 150 patients with polyneuropathy, ongoing burning pain was the most frequent and severe pain type, and its intensity correlated with laser-evoked potential attenuation [34]. Recently, a clinical and skin biopsy study showed that ongoing burning pain was strongly associated with regenerating sprouts, as assessed by GAP43 immunostaining [33].

In other NP conditions, such as brachial plexus injury, this type of pain results from anatomical denervation of second-order neurons that are exposed to local transmitters, giving rise to spontaneous firing [35].

Paroxysmal pain, described as electric shock-like or stabbing sensations, is associated with non-nociceptive Aβ fiber abnormalities [36,37]. In trigeminal neuralgia, a representative paroxysmal NP condition, focal compression of the trigeminal root causes demyelination of large myelinated fibers and increases their susceptibility to ectopic excitation and high-frequency discharges [38]. This peculiar mechanism of action explains the remarkable efficacy of voltage-gated sodium channel blockers, leading to the stabilization of hyperexcited neuronal membranes and the inhibition of repetitive firing. Identifying this pain quality in patients with other diseases affecting the peripheral nervous system may be crucial to select the most appropriate treatment.

Dynamic mechanical allodynia, the pain evoked by light moving tactile stimuli, is related to reactivity changes in central nociceptive neurons that increase their response to low-threshold Aβ afferent fibers [4]. According to recent neurophysiological and morphometric studies, allodynia in patients with peripheral neuropathy might also result from reduced mechanical threshold in hyperactive intraepidermal nociceptive afferents [39]. Accordingly, peripheral sensitization might play a role, together with second-order neuron sensitization to Aβ fiber input, in the development of allodynia.

Including the revised IASP grading system in future studies may reduce the heterogeneity of different pain causes by removing likely non-neuropathic ones. The revised algorithm should be used to reduce heterogeneity in clinical trials evaluating NP treatment and to improve the accuracy of effect estimates.

The grading system also makes it possible to test groups of patients with different pain types and whether they differ in terms of underlying pathophysiology or response to treatment.

## 5. Limitations

Although the revised IASP grading system criteria are crucial for guiding decisions on the level of certainty with which NP can be determined in an individual patient, some weaknesses in our current knowledge about NP can be identified. A definite diagnosis of NP is difficult to reach due to the lack of pathognomonic features characterizing this condition. Research aiming at identifying a gold standard for NP usually used the same NP criteria measuring the new introduced tools. This bias significantly hampered the validity of the studies. A meaningful area of research will be focused on using the present algorithm as a reference standard against which other neuropathic pain approaches should be systematically validated. Future studies assessing the grading system’s test–retest reliability and inter-rater reliability are needed.

Diagnostic tests are available only in specialized centers and require specific educational efforts.

In studies based on questionnaire surveys and patient interviews, only the level of “possible” NP can be reached. This limitation explains the exiguous number of studies identified in the present systematic literature search.

## 6. Conclusions

According to epidemiological studies, patients with peripheral neuropathy commonly suffer from NP as assessed by bedside clinical examination, neurophysiological testing, and IASP grading system criteria. Although specific NP qualities predominate in some peripheral nerve disorders (i.e., burning pain in brachial plexus lesions), the same types of NP usually develop across different aetiologies. Classifying the clinical symptoms in NP patients according to a mechanism-based approach might reduce the pathophysiological heterogeneity among study groups and is crucial to selecting the most appropriate treatment.

## Figures and Tables

**Table 1:** Frequency of neuropathic pain in peripheral neuropathy according to the International Association for the Study of Pain grading system criteria.

Author	Year	Patients (N°)	Condition	Patients with Neuropathic Pain (N°, %)
[5]	2018	314	Diabetic peripheral neuropathy	115, 37%
[6]	2020	126	Diabetic peripheral neuropathy	53, 42%
[7]	2017	107	Brachial plexus injury	60, 56%
[8]	2013	86 *	Thoracic surgery	66% **
[8]	2013	106 *	Breast surgery	68% **
[8]	2013	266 *	Hernia surgery	31% **
[8]	2013	142 *	THA/TKA	6% **
[9]	2008	23	Intercostobrachial neuropathy	5, 22%
[10]	2011	12	Pain after lymph node excision	10, 83%
[11]	2014	120	Chronic, non-malignant pain conditions	22, 18.3%
[12]	2013	152	Neck/upper limb pain	45, 30%
[13]	2012	2173	Any pain condition	639, 29.4%

* Median number of patients enrolled in the studies included in the systematic review. ** Relative prevalence. THA/TKA: total hip arthroplasty/knee arthroplasty.
